# In Situ Quantification of Bacterial Surface Charge at the Single-Cell Level for Modeling Transport under Electric Fields

**DOI:** 10.1021/acs.est.5c16185

**Published:** 2026-03-23

**Authors:** Shuai Wang, Feiyang Mo, Feifei Liu, Wei Wang, Xing Xie

**Affiliations:** School of Civil and Environmental Engineering, 1372 Georgia Institute of Technology, Atlanta, Georgia 30332, United States

**Keywords:** Bacteria transport, Electric field, Water disinfection, Charge, LEEFT

## Abstract

Locally enhanced electric field treatment (LEEFT) inactivates bacteria via charge-dependent transport into localized high-field regions, requiring resolution of surface-charge heterogeneity rather than population-averaged zeta potentials. Here, we develop a high-throughput single-cell tracking platform to quantify the effective surface charge at the individual-cell level. Using this approach, we reveal heterogeneity in effective surface charge spanning nearly 2 orders of magnitude under fixed medium conditions (pH 5.8) from −1.0 × 10^–18^ to −1.8 × 10^–16^ C. The least charged 10% of cells migrate at velocities up to an order of magnitude lower than the population average, while highly charged cells migrate much faster, enabling millimeter-scale transport within seconds. This contrast demonstrates that transport behavior is governed by the full effective charge distribution. While medium chemistry changes the overall charge level, with increasing pH shifting the surface charge from positive to negative values, we also found that the bacteria growth phase strongly modulates the width of the charge distribution. Together, these results not only validate the platform for resolving single-cell effective surface charge under operating electric fields but also provide distribution-resolved parameters that enable more accurate transport modeling and rational optimization of electric-field-based treatment systems.

## Graphical abstract

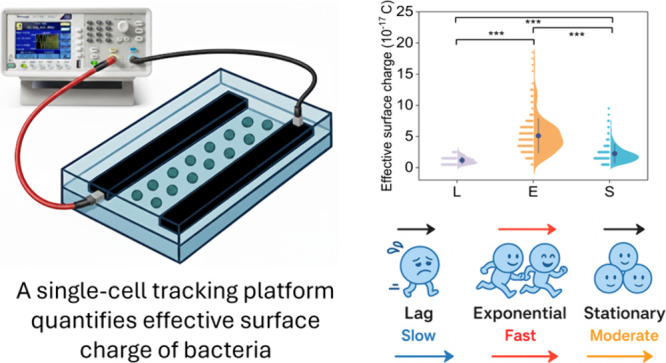

## Introduction

Bacterial contamination remains a critical concern in water treatment, healthcare, and food processing, threatening public health and destroying environmental quality.^1,2^ According to the World Health Organization, unsafe drinking water causes more than 500 000 diarrheal deaths each year. In response, electric field treatment (EFT) has emerged as a promising technique for water disinfection.^3–5^ Without chemical additives, EFT works by applying an external electric field to microbial suspensions, which induces electroporation (i.e., the formation of pores in bacterial cell membranes), thereby compromising membrane integrity and causing cell inactivation.^6,7^ Effective microbial inactivation by EFT typically requires high applied voltage (e.g., >10 kV) to ensure electroporation. Although effective, conventional EFT faces practical challenges, including high energy consumption (typically 10–100 kJ/L), extra cost that may be required to prevent overheating, and technical limitations to achieve a high-strength electric field without the risk of arcing at a large scale.^7,8^

To address these limitations, locally enhanced electric field treatment (LEEFT) has been developed.^7^ LEEFT employs micro- or nanoscale conductive structures (such as nanowires) to intensify the electric field via the *lightning-rod* effect, creating localized hot spots with a strong electric field.^9,10^ This strategy achieves bacterial electroporation at voltages of only a few volts, dramatically reducing energy demand. However, the efficacy of LEEFT requires bacteria to be transported into these intensified electric field regions.^11^ In a flow-through LEEFT reactor, only the microbes that migrate into the electric-field “hot spots” will be electroporated. Therefore, bacterial transport dynamics in the electric field become important for the LEEFT performance. A key factor governing bacterial transport under electric field is the electrostatic force, which is directly proportional to bacterial surface charge and electric field strength.^12^ Therefore, determining the accurate bacterial surface charge is essential in accurate modeling and optimization of LEEFT systems for practical applications.

Most studies still rely on ensemble-averaged techniques (most commonly, laser-Doppler electrophoretic light scattering (ELS) or related bulk zeta potential assays) to infer bacterial surface charge.^13–15^ In a standard ELS, scattered-light frequency shifts are converted into an ensemble-averaged electrophoretic mobility and corresponding zeta potential using Smoluchowski-type models that assume uniform particle response.^16^ Although apparent mobility distributions can be extracted from ELS data, these distributions are obtained through model-based inversion and reflect combined effects of optical weighting, particle size variability, and instrumental noise, rather than true single-cell charge heterogeneity.^17^ However, bacteria do not behave as a single mean population: van der Mei et al. directly resolved electrophoretic mobility distributions within single-strain populations, showing that averaged zeta potentials can overlook substantial cell-to-cell heterogeneity.^13^ This matters for modeling because population heterogeneity in surface charge causes individual cells to migrate at different speeds and directions, leading to deviations between predicted and observed transport.^18^ The real magnitude of cell-to-cell variation has to be characterized based on single-cell techniques, such as a high-resolution technique. For example, atomic force microscopy maps nanometer scale cationic and anionic patches on the surface of *Staphylococcus* and *Bacillus*, while scanning ion conductance microscopy resolves surface potential gradients across living *Escherichia coli* (*E. coli*) and *Pseudomonas* cells.^19,20^ However, these techniques require low throughput and immobilized cells, which cannot measure a single charge value under the operational fields. This leaves a clear knowledge gap in that the predictive LEEFT model lacks accurate single-cell effective surface charges measured under actual operating conditions. Measurements of single-cell charges and their distribution will be fundamental input parameters in the predictive bacteria transport and disinfection models in LEEFT systems.

In this study, we introduce a generalizable in situ, real-time, single-cell measurement framework to quantify the effective bacterial surface charge and its intrinsic heterogeneity in aqueous environments. The platform enables high-throughput tracking of individual bacteria under applied electric fields and directly extracts effective surface charge from electrophoretic motion using a force-balance formulation without relying on ensemble averaging or bulk inversion assumptions. This capability allows explicit resolution of charge distributions and low-mobility subpopulations that are inaccessible to conventional electrophoretic techniques or static single-cell probes. We demonstrate the utility of this framework using LEEFT. To achieve this, we employ high-resolution microscopy combined with advanced image-processing and tracking algorithms to capture trajectories of single bacterial cells under controlled electric field conditions. These trajectories are then used to compute the displacement, velocity, and eventually surface charge of each bacterial cell. By analyzing cells across lag, exponential, and stationary phases, we link the physiological state to electrical behavior and explain why growth phases differ in their charge distributions and motilities. This work establishes an in situ high-throughput platform that computes single-cell effective surface charge. Applied across growth phase and pH variability, this platform generates the model-based charge distributions important for future transport predictions and LEEFT optimization, ultimately improving real world microbial control and public health protection.

## Materials and Methods

### In Situ Observation Devices

A microscale device was developed to monitor real-time bacteria motion in an electric field. Specifically, the two parallel carbon electrodes were placed on a glass slide with a gap of 680 ± 10 μm. All measurements were performed in the channel midplane, approximately 340 μm away from either electrode surface, where wall-driven electroosmotic flow decays rapidly and does not dominate bacterial motion. The carbon electrodes were 2 cm long and 100 μm thick. Although the channel thickness (100 μm) is much larger than the bacterial cell size, only cells located within the microscope focal plane were tracked and analyzed. Cells that moved out of focus due to vertical (*z*-direction) motion were automatically excluded during image processing. The applied electric field was oriented in the imaging (*x*–*y*) plane, and the effective surface charge was calculated exclusively from in-plane electrophoretic velocities. As a result, potential out-of-plane motion does not affect the inferred charge values.

A coverslip was tightly attached to the top surface of the two electrodes to create a microchannel. Before each experiment, the device was flushed with 5% bleach and rinsed with sterile deionized water to eliminate residual microorganisms. An SP-300 Potentiostat (Biologic, USA) was used to control the electric field strength between the two carbon electrodes. The device maintained a constant current mode at 0, 50, 100, 200, 500, 1000, and 1500 nA for 10–30 s during bacterial motion experiments. Voltage and current data were recorded at 100 ms intervals for subsequent force-balance analysis. For comparative analysis, the 500 nA condition was selected as the final calculation. In this case, the applied electric field strength (*E*, 0.38 kV/m) was calculated by eq 1:

1 $$E=\frac{I}{\sigma A}$$

where *I* (5 × 10^–7^ A) is the applied current, σ (13.37 μS/cm) is the conductivity of the fluid, and *A* (2 × 10^–6^ m^2^) is cross-sectional area of the electrode.

The pH effect (from 3 to 10) was also tested under different electric field and growth state conditions.

### Bacteria Culture and Microscope Observation

*Staphylococcus epidermidis* (*S. epidermidis*, ATCC 12228) was used as a model bacterial strain in this study. It is a commonly used model bacteria strain in microbiology studies because its round and regular shape allows easier image processing for data acquisition.^21^ Due to its spherical geometry and lack of flagellar motility, confounding factors such as orientation-dependent hydrodynamic drag and active swimming components are effectively minimized. This simplification ensures that the observed heterogeneity in migration velocity is predominantly a function of the cell specific effective surface charge *q*, as defined in the force balance model. Bacterial cultures were grown in nutrient broth at 35 °C and harvested at three distinct time points representing different growth phases, 1.5 h (lag phase), 7 h (exponential phase), and 14 h (stationary phase), to systematically investigate the impact of the physiological state on surface charge. During the tracking experiments, cell division artifacts were avoided by utilizing short observation windows (10 to 30 s), which are significantly shorter than the bacterial doubling time. For each experiment, 4 mL of bacteria solution was washed three times with 10 mM phosphate-buffered solution (PBS, pH 7.4, and conductivity of 1592 μS/cm) by centrifuging at 4000*g* for 5 min and finally concentrated to 0.5 mL with deionized water (pH 5.8 and conductivity of 13.37 μS/cm). The concentrated bacteria solution (OD_600_ = 0.005) was then slowly injected into the microchannel and sat for 15 min to reach stability. The bacteria cells were randomly located in the microchannel, which was then loaded onto an inverted microscope for observation.

The microscope videos/images were captured through differential interference contrast (DIC) to track bacterial motion using a Zeiss inverted fluorescent microscope (Axio observer 7) connected to a CCD camera. The image capturing interval in videos was typically set as 200 ms, and the total recording time was 10–30 s. Three random regions were selected along the middle line inside the channels. All bacteria were located within the *x*–*y* plane, and their motion was tracked with and without an electric field.

### Image Processing and Single Cell Tracking

Image processing and single-cell tracking were implemented in Python 3.8 using OpenCV, NumPy, SciPy, and Pandas libraries. Image processing involved grayscale conversion, Gaussian blurring, adaptive thresholding, and morphological filtering to segment individual cells from DIC microscopy images. Valid contours were identified based on area (>50 pixels^2^) and minimum enclosing radius (>4 pixels, 1 pixel = 0.093 μm), and their centroids were used for tracking. The automated tracking algorithm further ensured data integrity by monitoring cell contour areas and centroids, excluding any trajectories that exhibited irregular morphological changes during the measurement period. A centroid-based matching algorithm with the Hungarian method was applied to assign detections across frames using a maximum association distance of 100 pixels (Figure S1). Instantaneous velocity was calculated from frame-to-frame displacement, and tracks were updated or removed based on continuity (Figure S2; full algorithmic details, parameters, and step-by-step examples provided in the SI).

For each retained track, cumulative displacement (in μm) was computed by summing the frame-to-frame Euclidean distances in the (*x*, *y*) plane using the calibrated spatial resolution. Instantaneous velocity for each bacterial cell at each time point was derived from dividing displacement by the interframe interval (0.2 s) and used to evaluate motility dynamics across time. To assess population-level heterogeneity, each data set was filtered to exclude zero-velocity artifacts and incomplete measurements. From the cleaned data set, a random subset of 30 unique cell tracks was selected for further statistical analysis. For each selected track, the average velocity was computed, representing that bacterium’s characteristic motility (all codes provided in the SI).

### Motion Analysis and Surface Charge Determination

The net surface charge of individual bacterial cells was calculated with a force balance model in a spatially uniform externally applied electric field. Each cell experiences an electrostatic force *F*_E_, a viscous drag force *F*_D_, and random Brownian motion in the tracking *x*–*y* plane. Because Brownian motion is nondirectional and its time-averaged displacement should be negligible compared with the deterministic electrophoretic drift, only the electrostatic and drag forces are included in the force balance. The experimental microchannel was designed to generate a spatially uniform electric field, ensuring consistent exposure across the field of view. In this configuration, negatively charged bacteria migrate toward the anode, while positively charged ones move toward the cathode. The net force can be expressed as eq 2:

2 $$F_{net}=F_{E}-F_{D}=Eq-6\pi \eta rv$$

where *q* (C) is the net surface charge on the cell, *E* is the applied electric field strength, 0.38 kV/m, η is the dynamic viscosity of the fluid, 10^–3^ Pa·s, *r* is the effective radius of the cell, 5 × 10^–7^ m, and *v* (m/s) is the real-time velocity. This balance of forces allows for a steady-state motion (*F*_net_ = 0) that directly links velocity to the averaged surface charge of individual cells.

### Mechanism Investigation

Membrane potential was measured using the voltage-sensitive dye Di-8-ANEPPS (Thermo Fisher Scientific), following a radiometric dual-excitation approach.^22^ Bacteria in different phases (lag, exponential, and stationary) were collected. Specifically, bacterial cells were incubated with 2 μM Di-8-ANEPPS in PBS for 20 min at 37 °C in the dark. After staining, cells were washed and resuspended in fresh PBS. Imaging was taken using a Zeiss inverted fluorescent microscope (Axio observer 7) equipped with two excitation filters (λ_1_ = 455 nm, λ_2_ = 511 nm) and a fixed emission filter (580–620 nm). Fluorescence images were sequentially acquired under the two excitation wavelengths by using identical exposure settings. Background-subtracted fluorescence intensities from each cell were measured at both excitations, and the ratio of intensities (*F*_511_/*F*_455_) was calculated for a minimum of 30 randomly selected cells per condition (Text S3).

Surface elemental compositions and chemical states of bacterial cells were analyzed using X-ray photoelectron spectroscopy (XPS). Cells harvested at different physiological stages (lag, exponential, and stationary) were collected and washed three times in PBS via centrifugation at 4000 rpm for 5 min to remove residual growth media. For surface analysis, intact dry cells were prepared following previous protocols using the freeze-drying method for 48 h.^23,24^ XPS measurements were performed using a Thermo Scientific K-Alpha XPS system equipped with a monochromatic Al Kα X-ray source (1486.6 eV). Spectra were calibrated by referencing the C 1s peak to 284.8 eV. Peak deconvolution of the C 1s region was carried out using Avantage software (v5.27).

## Results and Discussion

### Brownian Motion Analysis and Platform Verification

An in situ platform (Figure S3) was developed by connecting a potentiostat to a microchannel that contains bacteria. Prior to investigating bacterial behavior under electric fields, platform verification and baseline characterization of bacterial Brownian motion were performed. In the absence of an applied electric field, *Staphylococcus* cells exhibit purely diffusive behavior, as expected for Brownian motion. Based on the tracking video (Movie S1), we also plotted all trajectories (Figure 1a). Brownian motion trajectories reveal dozens of short-track segments, each colored by instantaneous velocity. Zoom-ins on five randomly selected tracks (all shown in Figure S4) highlight diverse behaviors. Some cells remain static, whereas others transiently explore larger displacements. Plotting the mean-squared displacement (MSD) versus time for all cells yields a near-perfect linear relationship (*R* ^2^ = 0.992, Figure 1b, validating the tracking accuracy of this device). From the slope (Slope = 4*D*),^25^ we extract a diffusion coefficient *D* = 0.16 ± 0.03 μm^2^/s, consistent with Brownian motion of 1 μm sized particles in water.^26–28^ A polar plot of step vectors (Figure 1c) demonstrates that, in the absence of external forces, movement directions are uniformly distributed.^29^ Color-coding by velocity confirms that velocity steps occur in all directions with equal probability, indicating no drift or directional bias in our imaging or analysis. The histogram of velocity (Figure 1d, only consider velocity magnitude here) shows a log-normal distribution with 80% of steps falling below 1 μm/s, producing submicrometer excursions over our frame interval. We also calculated the projected velocity on the *y* direction (Figure 1e, upward is positive and downward is negative). Although instantaneous measurements occasionally show *Y*-velocities approaching 3 μm/s, the averaged *Y*-velocity is only 0.004 μm/s, confirming that the motion is essentially nondirectional without net drift. Together, these data verify the reliability of our platform for quantifying bacterial transport and establish a robust baseline that, in the absence of an electric field, bacterial motion is purely diffusive and isotropic. This characterization is essential for subsequent calculations with an applied field.

**Figure 1:**
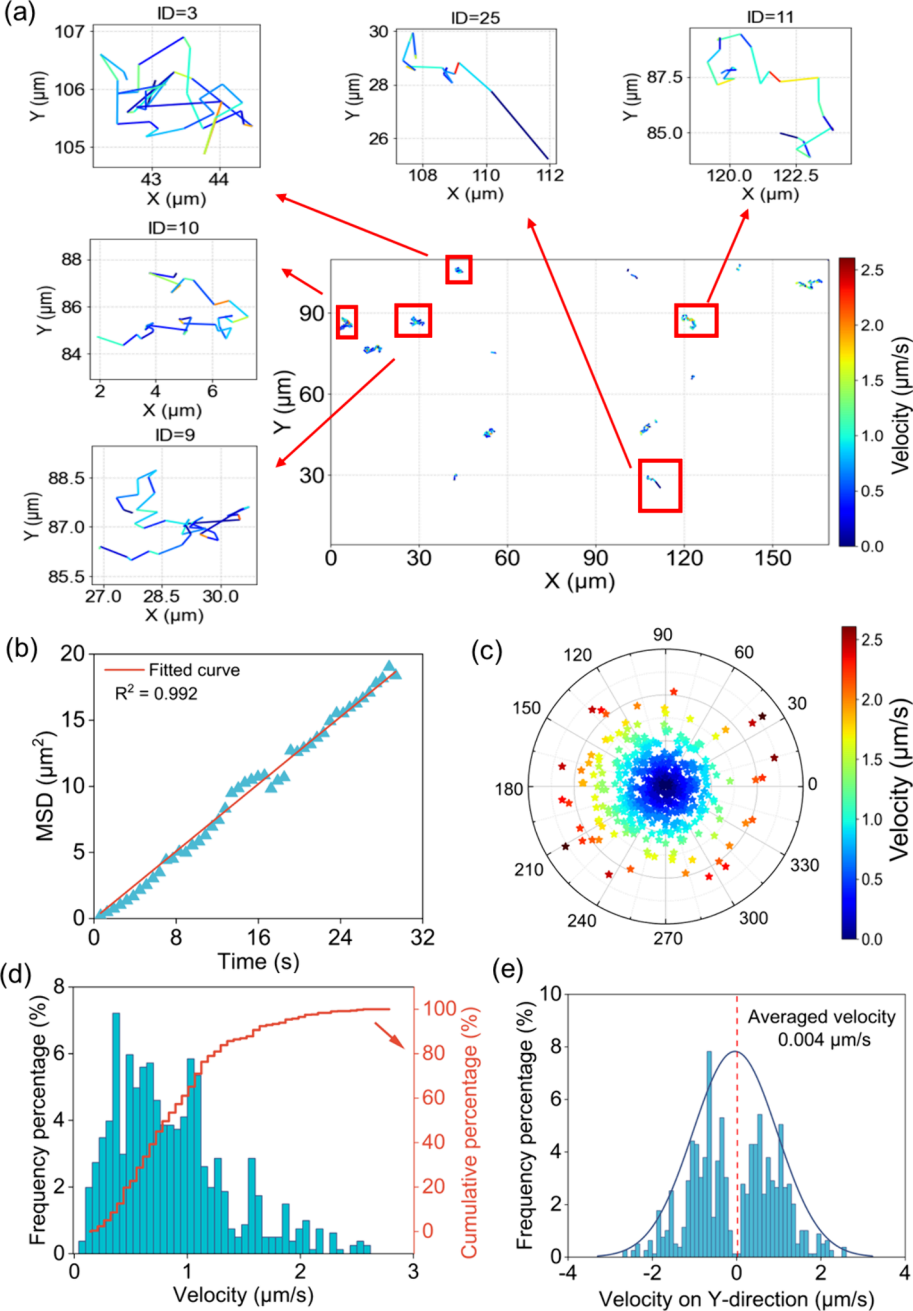
Field-off baseline validates unbiased single-cell motion and platform accuracy. (a) Two-dimensional trajectories of individual bacteria tracked over a 30-s interval. Inset shows magnified trajectories of five randomly selected cells, highlighting variability and randomness in displacement paths under conditions without electric field. (b) Mean-squared displacement (MSD) plotted as a function of time and fitted with a linear regression (*R* ^2^ = 0.992), confirming a purely diffusive (Brownian) motion pattern. (c) Polar plot (*n* = 898) shows bacterial velocities. The radial positions and color gradients represent the magnitude of velocity, while the angular positions indicate movement direction. The symmetric distribution confirms isotropic motion characteristic of Brownian diffusion. (d) The histogram of bacterial velocity of Brownian motion. (e) The Brownian velocity distribution on *Y*-direction (electric field direction in later experiments). Red dashed line shows the averaged velocity in the *y*-direction is 0.004 μm/s, which has insignificant effect on the overall electrical motion.

### Bacteria Motion under Electric Field Conditions

After verifying the in situ platform with Brownian motion analysis, we worked on bacterial response in electric field conditions. Bacteria in stationary phase were first chosen for our experiments because they exhibit minimal proliferation and active motility, reducing confounding effects on transport measurements.^30^ Figure 2a shows the in situ real time platform with electric field applied. All bacteria are suspended between the anode and cathode. The red-shaded region (0–10 s) in Figure 2b indicates the period during which the electric field was applied. During this period, bacteria exhibit rapid, directional displacement due to electrostatic force (Movie S2 and Movie S3). In contrast, the blue-shaded region (10–20 s) corresponds to the field-off condition during which bacterial movement significantly decreases and approaches zero, indicating that active transport is driven primarily by the electric field (Movie S4). Figure 2c illustrates the single bacterium microscopy figures from different time points (0, 2, 4, 6, 8 and 10 s). The color labels are locations at the previous frame, and the white line shows the moving path. The bacterium moves from anode to cathode under electric field conditions. With the tracked trajectory, we can further calculate the corresponding velocity and effective surface charge.

**Figure 2:**
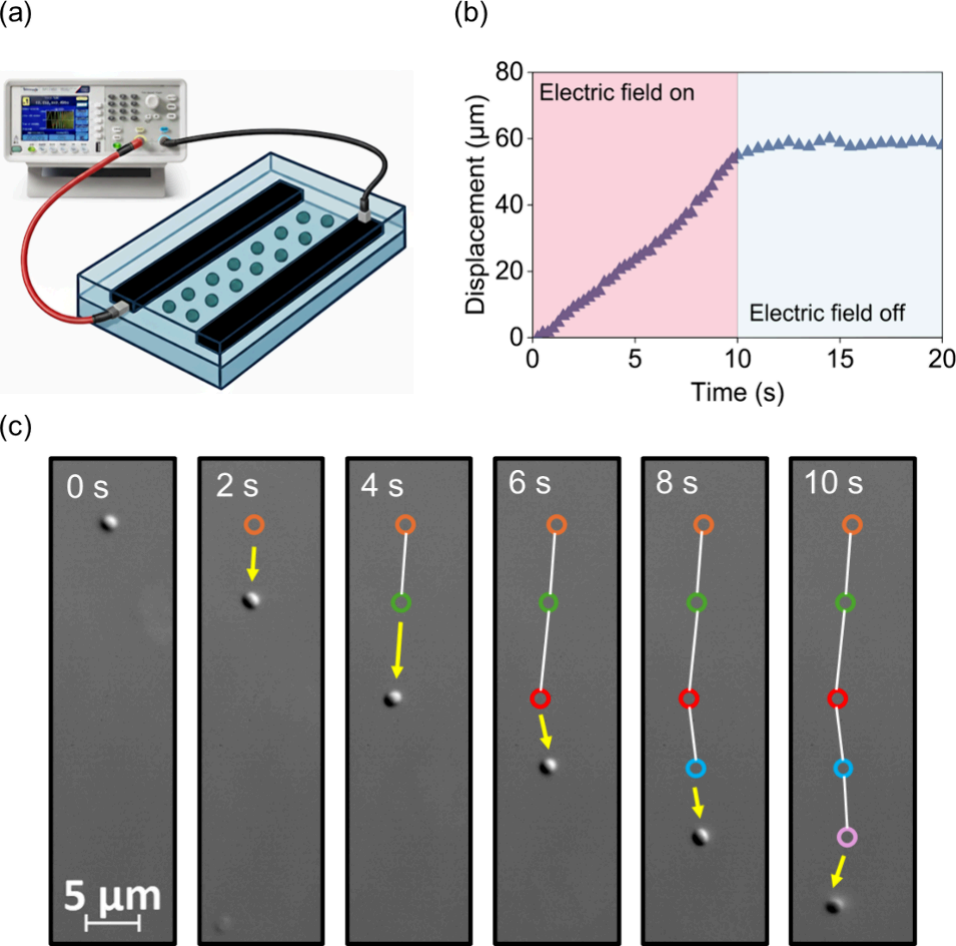
Field-driven bacterial migration in a microfluidic channel and single-cell movement. (a) Device schematic: a power source supplies current to two parallel electrodes patterned along a straight, bacteria-filled microchannel. (b) Displacement curve of bacteria in electric field. The initial linear increase in displacement corresponds to the electric field being turned on, indicating active bacterial motion. The plateau phase represents the stop of movement when the electric field is turned off. The transition between electric field “on” (pink region) and “off” (blue region) clearly demonstrates the influence of the electric field on bacterial displacement. (c) Bright field microscopy images of a single bacterium in an electric field of 0.38 kV/m, showing the bacterium moves toward the cathode. Each circle represents the previous location.

With trajectory determination, we further calculated the velocity from the trajectories (*n* = 429 cells). All of the bacteria are suspended in solution with pH 5.8 and an electric field strength of 0.38 kV/m. Figure 3a shows the velocities for 30 randomly selected individual cells. A red dashed line indicates the overall average velocity of 6.29 μm/s calculated from all cells tracked. As mentioned above, the average *Y*-direction velocity of Brownian motion is 0.004 μm/s, which can be negligible compared to the motion under electric field conditions. However, considering the bacterial movement in each time interval, Brownian motion cannot be negligible. Frame velocities show that individual bacteria fluctuate between slow and fast motion during their trajectory (Figure S5). The temporal fluctuations in their directional movement are significantly contributed by Brownian motion at each time point. Such thermal fluctuations are inherent in microscale biological systems and tend to randomize cell orientation and trajectory over short time scales. However, in the presence of an external electric field, this randomness is partially suppressed as the field exerts directional force that biases overall movement.^31^ Additionally, collisions between bacteria along the *Y*-axis (i.e., when faster cells encounter one another) may transiently alter their velocity.^32^ However, this variability had little effect on the overall motion velocity and direction of the electric field. So, electrostatic force imposed by the electric field dominates over stochastic and intercellular interactions, effectively guiding the net bacterial motion despite local interaction. Figure 3b shows the 2D trajectories of all tracked cells. These trajectories are color-coded according to velocity, making it possible to visually distinguish between different trajectories (displacements over time shown in Figure S6). The trajectories are almost aligned with the *Y*-direction, which indicates that all bacteria are subjected to electrostatic force and move along with the electric field direction. This visualization clearly shows significant differences among cells: some cells follow longer trajectories (>75 μm), indicating higher velocity, while others trace much shorter paths (30–40 μm).

**Figure 3:**
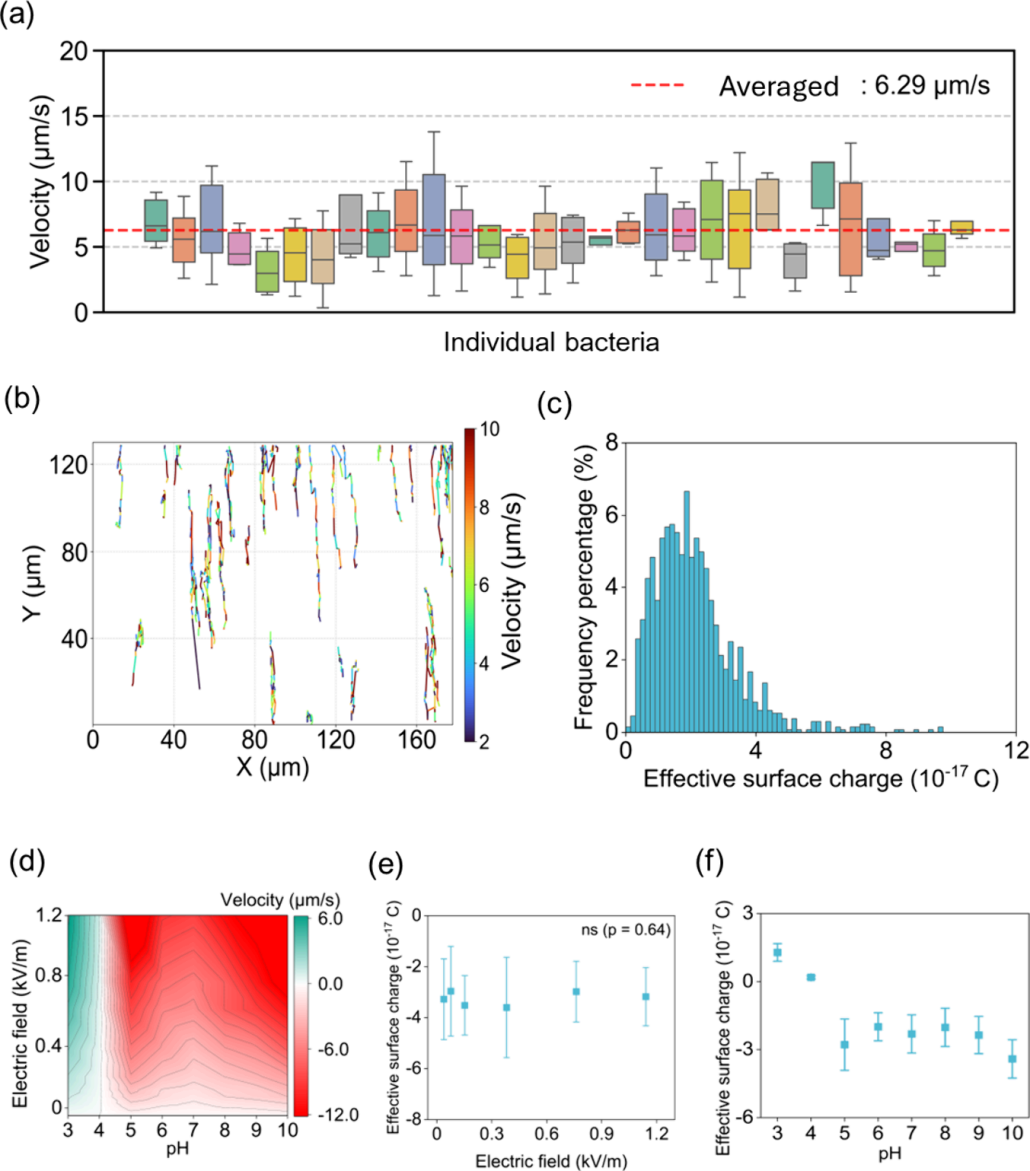
Single-cell heterogeneity of field-driven motility and calculated effective surface charge. (a) Box plots of velocities for 30 representative cells with the red dashed line indicating the overall average velocity (6.29 μm/s, calculated from 429 cells). Boxes show the interquartile range (IQR), whiskers extend to 1.5 × IQR, and horizontal bars represent medians. (b) 2D trajectories of all tracked cells, color-coded by velocity to highlight intercellular variability. (c) Histogram of all bacteria effective surface charges calculated from eq 2 (*n* = 429), revealing a broad distribution and underscoring the inherent heterogeneity in bacterial motility. Calculated charge is negative, and the figure here only considers the magnitude. (d) Average cell velocity distribution under different pH values (3–10) and electric fields (0–1.2 kV/m). Warmer colors denote motion toward the anode (negative velocity). Colder colors denote motion toward the cathode (positive velocity). White color denotes isoelectric point. (e) Effective surface charge of cells under different electric field strengths (0–1.2 kV/m). Pearson correlation analysis (*p* = 0.64) indicated no significant relationship between electric field and surface charge, suggesting that the charge remained statistically constant across the tested range. (f) Calculated effective surface charge versus pH, obtained from eq 2.

### Effective Surface Charge Determination

The heterogeneous motion observed among individual bacteria cells under electric fields raises a fundamental question. What factors drive this variability in the transport behavior? From eq 2, the outer electric field, fluid viscosity, and hydrodynamic radii are essentially the same for all cells in the suspension, so the only term that can generate the observed spread in velocities is the surface charge *q*. Across thousands of bacteria tracked in the experiments, we averaged each bacterium’s velocity and converted it to the effective surface charge from eq 2. The resulting population distribution is shown in Figure 3c. Under these working conditions, bacteria are negatively charged, and the figure here considers only the magnitude of the calculated charges (positive values shown). The histogram demonstrates a broad pattern: most cells possess moderate negative charges between approximately −1.1 × 10^–18^ and −9.7 × 10^–17^ C with frequencies peaking near −2 × 10^–17^ C. A long tail extends toward higher charge values with a small percentage of cells exhibiting charges exceeding −8 × 10^–17^ C. Together, the data show that the wide spread of velocities directly indicates the broad distribution of cell-specific surface charges: cells with larger negative charges experience stronger electrostatic forces and migrate faster, whereas less charged cells move more slowly.

It is important to emphasize that the surface charge determined here does not represent the absolute surface charge of each bacterium but, rather, the “effective surface charge” experienced by a bacterium moving through an aqueous medium under an applied electric field.^33^ From a theoretical perspective, this behavior is well described by the soft-particle electrophoresis framework, which treats bacterial cells as permeable charge-regulated entities rather than rigid colloids. Because bacterial envelopes behave as soft, permeable polyelectrolyte layers (e.g., lipopolysaccharide and capsule), the hydrodynamic slip plane lies within this layer. Only charge outside that plane drives electrophoresis, while charge within is immobilized and screened by penetrating counterions, resulting in a much smaller effective charge estimated from mobility.^34,35^ The absolute bacterial surface charge is governed by the surface chemical groups, and previous studies showed that the actual bacterial surface charge is −1.9 × 10^–13^ to −2.6 × 10^–13^ C.^36,37^ In practical disinfection applications, attention should be directed toward the effective surface charge, which ultimately governs migration and bacterial response in LEEFT. Although the present study focuses on a Gram-positive model bacterium (*S. epidermidis*), the measurement principle is independent of Gram classification and relies on operando tracking of cell motion under applied electric fields. Structural differences in Gram-negative envelopes, such as the presence of an outer membrane and lipopolysaccharides, are therefore expected to modulate the magnitude and heterogeneity of the effective surface charge rather than the applicability of the platform itself. Extending this framework to Gram-negative bacteria represents an important and promising direction for future work, as it would enable a systematic comparison of how envelope architecture influences charge regulation and transport behavior under electric fields.

Since the ionization of outer-surface groups varies with pH, we next quantify the effects of pH and field strength on both the migration velocity and effective surface charge. Figure 3d shows the velocity under different pH and electric field conditions. Velocity increased monotonically with electric field at all pH values tested (0–1.2 kV/m). At pH = 3, the positive velocities (green color label) indicate net positive charge; as pH rises past the isoelectric point (around pH 4), the sign reverses and cells become negatively charged (red color label), establishing pH as a primary determinant of charge and mobility. Then, we controlled pH as 5.8 (DI Water) and plotted the velocity versus electric field. No significant correlation was found between the electric field strength and the effective surface charge (*p* = 0.64), indicating that the surface charge remained constant across the tested range of electric fields (Figure 3e, 0–1.2 kV/m). We further calculated the effective surface charge under different pH values (Figure 3f, electric field = 0.38 kV/m). Charges are positive at pH 3–4, reach a minimum at pH 4 (0.1 × 10^–17^ C), become increasingly negative from pH 4 to 6, and then plateau at pH ≥ 6 (−3.4 × 10^–17^ C). Prior studies emphasized pH as the primary factor for tuning bacterial surface charge,^38^ but our single-cell map (Figure 3c) shows that the population’s physiological composition co-determines the effective surface charge.

Importantly, pH represents only one dimension of the broader chemical environment that regulates the bacterial surface charge. In natural and engineered water systems, variations in ionic composition and the presence of natural organic matter are also expected to influence surface charge regulation and membrane potential through electrostatic screening, ion association, and interactions with charged macromolecules. Such effects would be expected to shift not only the mean effective surface charge but also its distribution across the population under applied electric fields. While the present study focuses on controlled conditions to establish a clear mechanistic baseline, extending the framework to chemically complex waters will provide further insight into how water chemistry modulates charge heterogeneity and, consequently, transport behavior in electric-field-based processes.

### Growth Phase Discrepancy

Bacteria exhibited varied physiological states across growth phases, and such variability becomes even more important under real environmental conditions where nutrient availability and growth stress fluctuate, influencing both surface properties and motility.^39–41^ Building on our earlier finding that single-cell surface charge variability drives significant differences in motility, we further examined the bacterial motility across different growth phases. The growth curve of *Staphylococcus* (Figure S7) identifies distinct phases: lag phase (L), exponential phase (E), and stationary phase (S). Figure 4a shows single-cell velocities measured during the lag phase tightly around 2–5 μm/s with an average velocity of 3.48 μm/s (Movie S5). There is only a small fraction of cells exceeding 6 μm/s, indicating relatively uniform motility at this growth stage. The corresponding two-dimensional trajectories (Figure 4b) show mostly short moving paths under this condition. Figure 4c shows the effective surface charge calculated by using the same method. Most cells carry effective surface charges between −1.0 × 10^–18^ C and −3.0 × 10^–17^ C, peaking near −1.5 × 10^–17^ C. In contrast, exponential phase cells (Movie S6) display much broader velocity distributions (Figure 4d) with velocity ranging from near 1 μm/s up to 16 μm/s and an overall average of 8.46 μm/s. Whiskers span 1–20 μm/s for individual bacteria, highlighting pronounced intercellular variability compared with the lag and stationary phases. At this condition, bacteria have a long uniform moving path in the *x*–*y* plane and align with the *y* direction (from anode to cathode in Figure 4e). The exponential phase charge histogram (Figure 4f) shows a wider spread with charges from −8.5 × 10^–18^ C to −1.8 × 10^–16^ C, and the bulk of the population lie between −2.0 × 10^–17^ C and −6.0 × 10^–17^ C.

**Figure 4:**
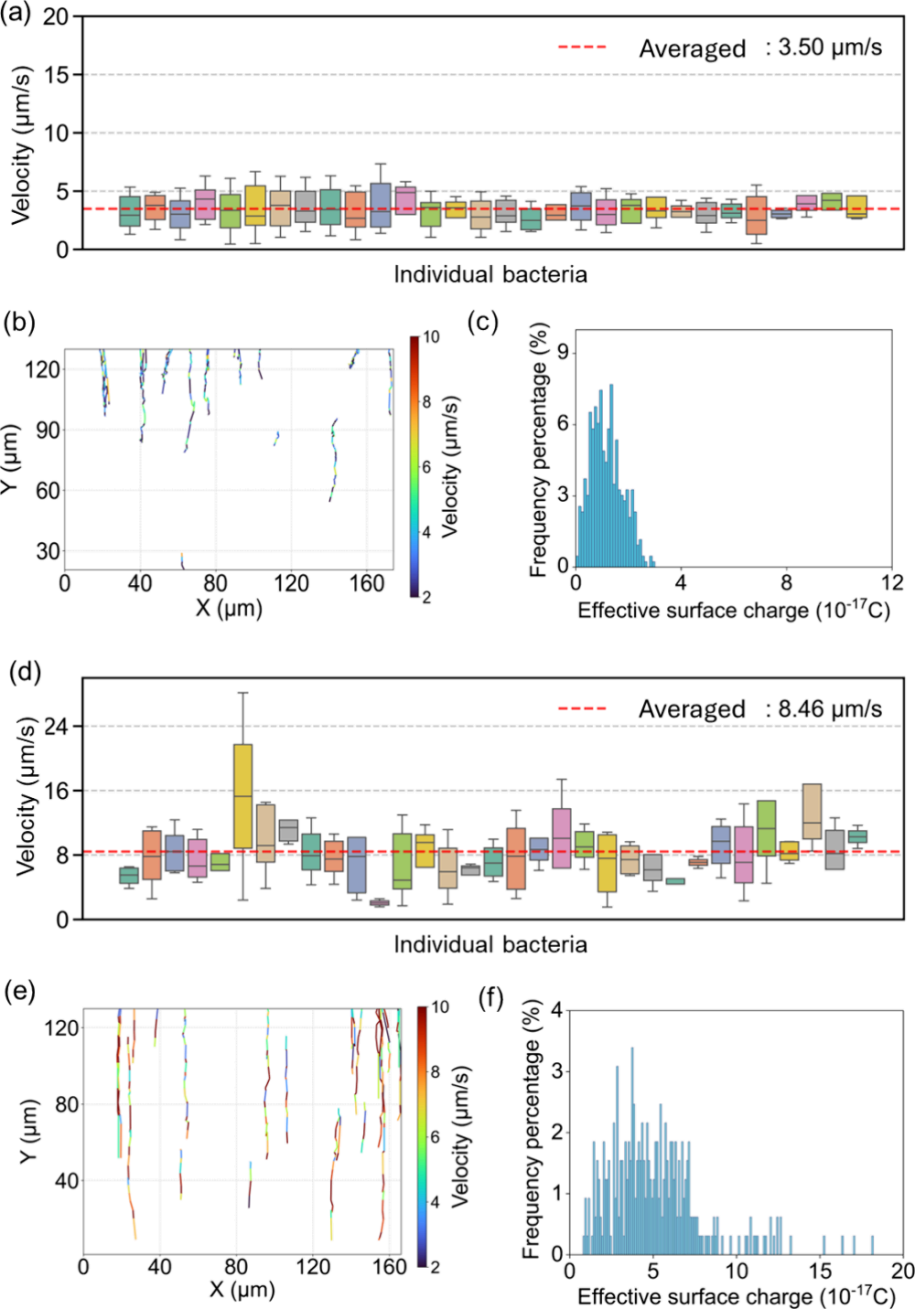
Bacterial motility and charge distributions in lag phase (a–c, *n* = 320) and exponential phase (d–f, *n* = 324). (a and d) Box plots (30 randomly selected cells are shown) of single cell velocity measured during the lag phase; each box represents the interquartile range (IQR) of velocities for an individual cell with whiskers extending to 1.5 × IQR. The red dashed line indicates the overall average velocity of all cells. (b and e) 2D trajectories of all tracked cells, color-coded by velocity to highlight interindividual variability. (c and f) Histogram of effective surface charges for cells in the lag phase and exponential phase. Bacteria are negatively charged in these experiments; the figure shows absolute charge values (|*q*|) for clarity.

Mechanistically, this discrepancy arises from dynamic remodeling of the cell envelope during exponential growth, which changes the density and the exposure of surface anionic groups. In *Staphylococcus*, the biosynthesis of wall teichoic acids and anionic membrane lipids increases substantially during rapid growth, exposing additional negatively charged phosphate and carboxyl groups and thereby elevating both the mean surface charge and its variability.^42^ Consistent with this interpretation, Hayashi et al. demonstrated through soft-particle electrophoresis that bacterial electrokinetic characteristics vary systematically with growth phase, confirming that physiological state modulates the effective surface charge.^43^ Similarly, Hong and Brown demonstrated that the charge-regulated bacterial envelope undergoes dynamic acid–base speciation, producing broad ζ-potential distributions within a single growth state.^36^ Flow cytometry further confirmed that capsule expression in *S. aureus* is heterogeneous within the same culture, leading to differences in surface anionic shielding between cells.^44^ Beyond *Staphylococcus*, comparative studies on lactic acid bacteria found culture to culture variability and disrupted S-layers even in exponential cells, reinforcing the generality of intraphase variation.^45^ At a broader scale, phenotypic heterogeneity and stochastic gene expression are recognized as intrinsic features of microbial populations, maximized during rapid growth.^39^ While effective surface charge is shown here to be a key driver of transport variability, it is important to recognize that effective charge itself is not a fixed cellular property. Rather, it reflects an integrated outcome of multiple physiological and interfacial processes at the single-cell level. In addition to growth phase dependent envelope remodeling, intrinsic features of microbial populations, such as stochastic gene expression and phenotypic heterogeneity, can lead to cell-to-cell differences in the abundance, accessibility, and organization of charged surface components.^39,46^ These variations regulate charge dissociation, electrostatic screening, and ion association at the cell–water interface, resulting in heterogeneous effective surface charge and, consequently, variable transport behavior under applied electric fields.

Consistent with these biochemical differences, our statistical analysis of effective charge values across the three phases (−1.18 × 10^–17^, −5.12 × 10^–17^, and – 2.77 × 10^–17^ C for L, E, and S; *p* < 0.001) confirms a significant and systematic phase dependence (Figure 5a). Lag phase cells show a narrow, low charge distribution. Exponential phase cells exhibit a broader distribution shifted toward more negative charges. Stationary phase cells lie between these two phases. Figure 5b shows the comparison of bacteria cells in different growth phases via DIC and fluorescence imaging (more images shown in Figure S8). Fluorescence microscopy using Di-8-ANEPPS, a voltage-sensitive dye, takes advantage of its dual-emission properties: when the membrane is hyperpolarized, the dye preferentially emits in the green channel, whereas depolarization shifts the emission toward the orange channel.^22^ In the lag phase (Figure 5b-L), low metabolic activity and stable ionic gradients produce uniformly hyperpolarized membranes, yielding dominant green fluorescence and the lowest orange/green ratio (Figure 5c). Upon entry into the exponential phase (Figure 5b-E), rapid synthesis of negatively charged lipids and lipopolysaccharides partially depolarizes the membrane, shifting Di-8-ANEPPS emissions strongly toward orange and driving the orange/green ratio to its maximum. Single-cell tracking data (Figure 4) show that cells in this phase exhibit the highest average velocities and the greatest cell-to-cell variability. In the stationary phase (Figure 5b-S), slow metabolism and surface remodeling allow partial repolarization, resulting in mixed orange and green emissions and an intermediate orange/green ratio. Correspondingly, the velocity is moderate and becomes more uniform (Figure 3). Thus, the fluorescence results indicate that membrane polarization varies systematically with growth phase (maximal depolarization in exponential cells, intermediate in stationary, and minimal in lag), consistent with the phase-ordered effective surface charge distributions.

**Figure 5:**
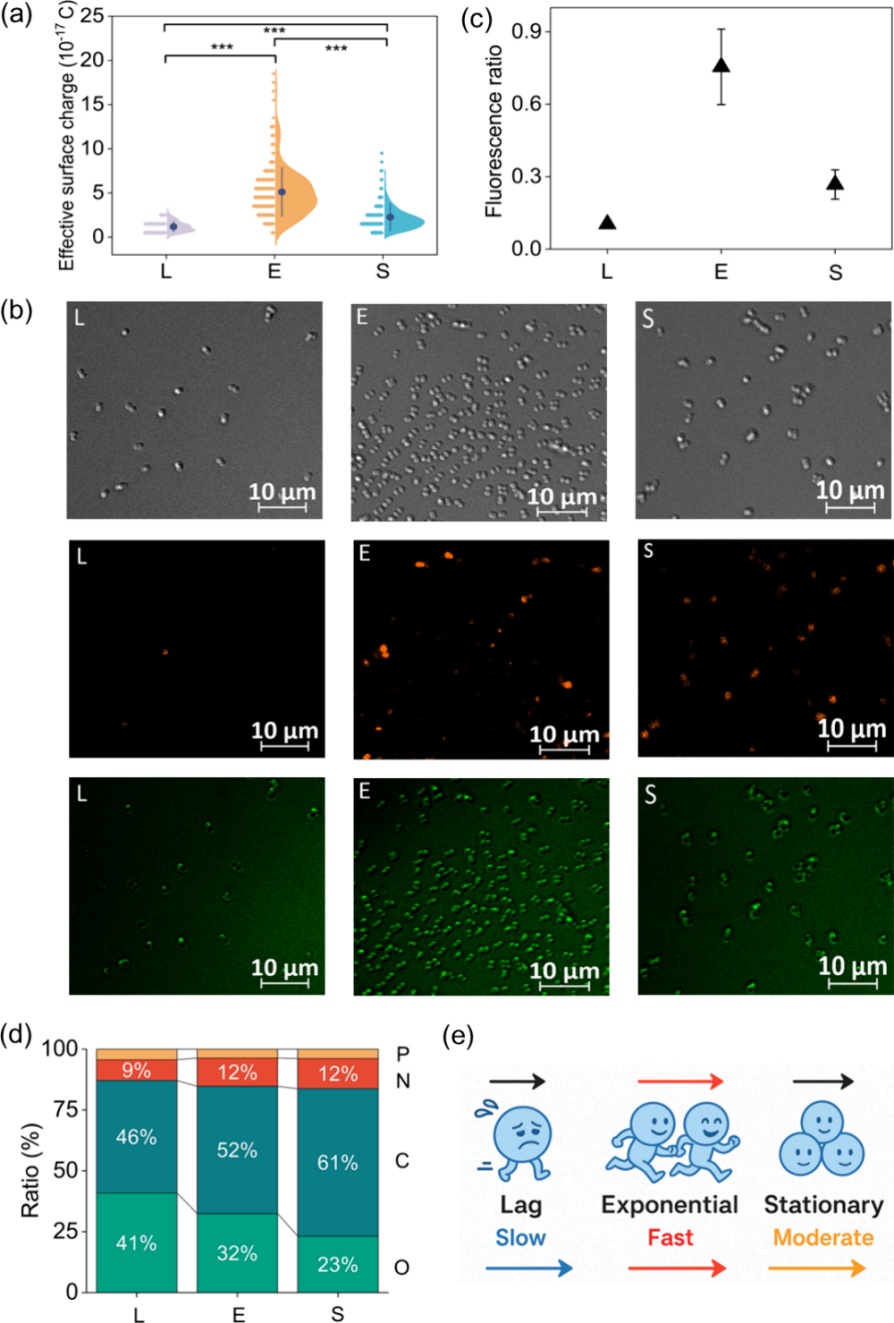
Physiological state modulates effective surface charge and transport. (a) Plots of effective surface charge per cell measured in each growth phase (L is lag, E is exponential, and S is stationary). Only charge magnitude is considered (all charges are negative). Pairwise comparisons (L vs E, L vs S, and E vs S) are indicated as highly significant (***, *p* < 0.001, one-way ANOVA). (b) Representative DIC (top) and fluorescence images (middle and bottom) of Di-8-ANEPPS-labeled bacteria in L, E, and S phases. Orange (middle) and green (bottom) channels correspond to depolarized and hyperpolarized membrane states, respectively. (c) Quantification of orange/green fluorescence intensity ratios for different growth phases, showing peak membrane depolarization during exponential phase (*n* = 30). (d) Elemental composition of membrane surface for bacteria in different growth phases based on XPS analysis. (e) Cartoon schematic illustration for bacterial transport under different growth phases.

The phase-dependent elemental composition revealed by XPS (Figure 5d and Figure S9) provides chemical insight into the observed variation in effective surface charge across growth stages. From the lag to exponential phase, the relative phosphorus contribution increases from 9% to 12%, indicating enhanced exposure of phosphate-containing components such as wall teichoic acids and membrane phospholipids during active cell growth.^43,47^ These anionic polymers are a major source of negative charge in Gram-positive bacteria and are known to strongly influence electrokinetic behavior through acid–base dissociation and counterion association.^36^ The carbon fraction increases (46% to 52%), reflecting intensified biosynthesis and turnover of envelope polymers during exponential growth. In the stationary phase, carbon content further increases to 61% while oxygen decreases to 23%, suggesting surface restructuring and partial shielding of highly ionizable functional groups, consistent with reduced accessibility of charged groups.^45^ Together, these results indicate that growth stage dependent surface chemistry governs both the magnitude and heterogeneity of effective surface charge, thereby directly impacting electrophoretic transport behavior. Because transport into high-field zones in LEEFT is approximately proportional to the effective charge (Figure 5e), the growth phase greatly affects the effective charge and shows the comparable effect of pH. Incorporating the growth state alongside pH into LEEFT models and operations should improve prediction and control.

### Environmental Implications

The platform developed in this work for effective surface charge measurement provides a new capability for detailed surface charge determination in real working scenarios. Conventional measurements of bacterial zeta potential or surface charge rely on bulk assays that provide a single mean value, thereby ignoring the population level diversity. In contrast, our approach quantifies the effective charge of individual cells under applied fields, allowing charge heterogeneity to be resolved in real time. While the present study utilizes *S. epidermidis* as a geometric controlled model due to its spherical shape, the developed framework is broadly applicable across diverse microbes. The measurement principle can be extended to a broader range of microbial morphologies, including rod-shaped or flagellated bacteria such as *E. coli*, by incorporating morphology specific drag tensors and active motility terms into the existing force balance model. This versatility enables comparison not only between different species but also among growth states and even complex environmental samples, for example, mixed microbial communities from natural waters and sediments. At present, the system represents a prototype measurement platform. Future integration with automated microfluidics and AI-assisted image analysis may further increase throughput and enable scalable identification and classification of bacteria or particles in heterogeneous environmental samples, although such extensions are beyond the scope of this study. Future studies can use it to build a database of single-cell effective charges across diverse bacteria and environmental samples. By providing single-cell charge parameters, the platform creates a foundation for more accurate transport models in engineered and natural systems from water treatment to biofilm colonization.^48^

In water treatment applications, these data provide direct insights into LEEFT design. Because bacterial inactivation occurs only within localized high-field regions, the overall disinfection performance is affected by the fraction of cells that are successfully transported into these regions rather than by the average response. In practice, achieving complete (100%) delivery of all cells is neither realistic nor necessary; instead, engineering targets typically aim to ensure that a defined majority of the population (e.g., ≥99.99%) reaches the high-field zones under given operating conditions. Quantifying the full distribution of effective surface charge therefore enables a more accurate description of bacterial transport and a more reliable prediction of log-removal efficiency. Identifying the weakly charged bacteria allows process parameters, such as electric field waveform, voltage amplitude, and hydraulic residence time, to be optimized to ensure sufficient transport of this subpopulation. In addition to tuning LEEFT operating parameters, water properties that influence bacterial surface charge, including pH, ionic composition, and natural organic matter, should be considered as complementary design levers. Together, these strategies enable a higher and more robust disinfection performance while minimizing energy consumption in practical water treatment systems.

## Supplementary Material















## Notes

Texts S1–S3: the complete image-processing pipeline, data analysis, and dual-channel fluorescence analysis; Figures S1–S3: image analysis workflow, object detection examples (Hungarian association method), and the in situ microchannel platform with the force-balance model; Figures S4–S6: two-dimensional Brownian trajectories, velocity heat maps for 30 representative cells, and corresponding 3D displacement–time–ID plots: Figures S7–S9: growth-phase references, including the *Staphylococcus epidermidis* growth curve, representative DIC micrographs for lag-, exponential-, and stationary-phase cells, and XPS survey spectra with C 1s deconvolution confirming surface chemical-state changes (PDF)
Movie S1: Brownian motion under zero field (AVI)
Movie S2: positively charged cells migrating to the anode (AVI)
Movie S3: negatively charged cells migrating to the cathode (AVI)
Movie S4: representative motions of lag-phase cells (AVI)
Movie S5: representative motions of exponential-phase cells (AVI)
Movie S6: representative motions of stationary-phase cells (AVI)
