# Supplementary material for: In Situ Quantification of Bacterial Surface Charge at the Single-Cell Level for Modeling Transport under Electric Fields
Source: Environ Sci Technol. 2026 Mar 23;60(13):10309–19. doi: 10.1021/acs.est.5c16185 (PMC13063416; doi:10.1021/acs.est.5c16185)
Supplement: Supplementary file 1 [file es5c16185_si_001.pdf]

In situ Quantification of Bacterial Surface Charge at the Single-Cell Level for Modeling Transport under Electric Fields

Shuai Wang [a], Feiyang Mo [a], Feifei Liu [a], Wei Wang [a], Xing Xie [a, *]

[a] *School of Civil and Environmental Engineering, Georgia Institute of Technology, Atlanta, Georgia 30332, United States.*

Corresponding author: Xing Xie (email: xing.xie@ce.gatech.edu)

This supporting information contains 25 pages, 3 supplementary notes, 9 supplementary figures, 2 supplementary codes and 6 supplementary videos.

Table of contents

1. **Supplementary notes**

   Supplementary text 1 Image processing and single cell tracking

   Supplementary text 2 Data analysis

   Supplementary text 3 Fluorescence analysis

2. **Supplementary figures**

   Supplementary figure 1 Image-analysis workflow.

   Supplementary figure 2 Detection examples (Hungarian method).

   Supplementary figure 3 In-situ microchannel platform & force balance.

   Supplementary figure 4 All bacterial Brownian motion trajectories in 2D scale.

   Supplementary figure 5 Heat map for 30 cells revealing the phase-dependent velocity.

   Supplementary figure 6 3D displacement–time–ID figure of 30 cells.

   Supplementary figure 7 Growth curve of *Staphylococcus epidermidis* (L/E/S).

   Supplementary figure 8 Representative DIC micrographs (L/E/S).

   Supplementary figure 9 XPS survey & C 1s deconvolution (L/E/S).

3. **Supplementary codes**

   Supplementary code 1 Track detection

   Supplementary code 2 Trajectory analysis

Text. S1 Image processing and single cell tracking

The following section presents the comprehensive computational methods provided in the main text. All image processing and bacterial tracking workflows were developed using Python 3.8.10 on a Windows 11 system equipped with an AMD 5000 series CPU. Key libraries included OpenCV 4.5.5 for image processing, NumPy 1.21.6 for numerical operations, SciPy 1.7.3 for mathematical routines, Pandas 1.3.5 for structured data handling, and scikit-learn 1.0.2 for auxiliary support.

Videos were recorded at 5 frames per second using DIC microscopy to capture bacterial motion. Each frame was first converted to grayscale to simplify intensity manipulation, and then denoised with a Gaussian blur kernel to reduce spatial noise and normalize background intensity. The Gaussian blur parameters were selected empirically to retain edge contrast while suppressing high-frequency background artifacts, especially in unevenly illuminated regions.

Segmentation was performed using adaptive Gaussian thresholding with a block size of 11 and constant subtraction value $C = 2$. These values were chosen after comparative testing across block sizes of 7, 11, and 15 and C values of 0, 2, and 4. The 11/2 combination was found to yield the best trade-off between sensitivity and specificity, minimizing false positives to 5% when benchmarked against manual annotations across 50 frames. Following thresholding, binary masks were refined using morphological opening with a 5×5 elliptical kernel. This step effectively eliminated speckle noise and small artifacts while preserving the integrity of true cell contours; it removed over 95% of noise objects while retaining most biologically relevant regions, based on a manual validation dataset.

From the cleaned binary images, contours were extracted, and size filtering was applied to exclude non-bacterial artifacts. Only contours with an area exceeding 50 pixels^2 (~0.43 μm^2) and a minimum enclosing circle radius greater than 4 pixels (~0.37 μm) were retained. These thresholds were established based on expected bacterial dimensions under the imaging setup (1 pixel = 0.093 μm) and were validated by visual review.

For each valid contour, the centroid was computed using image moments and used as the positional marker for the corresponding bacterium in that frame. A custom tracking algorithm was then applied to link

centroids across consecutive frames. Initially, centroids detected in the first frame were assigned unique track IDs. For each subsequent frame, a cost matrix of Euclidean distances was computed between current and previous centroids. The Hungarian algorithm was used to solve the optimal assignment problem. Only pairings with distances ≤ 200 pixels were accepted, a value chosen based on manual analysis of real trajectories.

The throughput of the platform is defined by the number of individual bacterial trajectories that can be simultaneously acquired and analyzed within a single experiment. Under typical experimental conditions, bacterial suspensions were adjusted to yield approximately 50–200 cells within a 120 × 160 μm^2 field of view at any given time. Videos were recorded at a temporal resolution of 200 ms per frame for 10–30 s, resulting in 50–150 frames per experiment.

Each experiment therefore generates on the order of 10^3–10^4 individual displacement and velocity data points, depending on cell density and observation time. Trajectories are identified and linked across frames using an automated image-processing pipeline implemented in Python, which includes background subtraction, object detection, and frame-to-frame association based on the Hungarian algorithm. This automated workflow eliminates the need for manual tracking and enables rapid processing of large datasets, allowing statistically meaningful distributions of velocity and effective surface charge to be extracted from a single experimental run.

Unmatched detections were assigned as new tracks, while existing tracks were terminated if they failed to receive a match for more than 10 consecutive frames. These two rules effectively prevented fragmentation and over-linking, ensuring temporal coherence. For each matched detection, displacement was calculated in micrometers by multiplying pixel shift by 0.093 µm, and instantaneous speed was determined by dividing displacement by the frame interval (0.2 s). Angular changes between consecutive vectors were also computed and recorded. To analyze bacterial motion, only tracks with at least 40 valid data points were retained for downstream processing. Some of the bacteria are missing due to the collision and fluctuation at some frames so not all the motions are in full trajectories. All final data, including frame number, track ID, X/Y position, speed, and direction were saved for further quantitative analysis and visualization. The

pipeline offers a robust and reproducible framework for tracking bacterial motion at the single-cell level with minimal manual intervention.

Text. S2 Data analysis

To quantify and classify bacterial motility, we analyzed only complete tracks with at least 30 timepoints, ensuring sufficient temporal resolution to capture representative behavior. The frame rate of 5 frames/s was used to convert frame indices to elapsed time (0.2 s per frame), and spatial displacement was computed using the calibrated pixel size (0.093 μm/pixel). For each bacterium, cumulative displacement was obtained by summing frame-to-frame Euclidean distances in the (X, Y) plane. Instantaneous speeds were then calculated for each frame by dividing displacement by the inter-frame time interval. This generated a high-resolution time series of motility behavior per cell.

To minimize noise and artifacts in the population-level dataset, we excluded data points with zero or undefined speeds, typically arising from frame loss, temporary segmentation failure, or cell pausing beyond the detection threshold. A random subsample of 30 unique track IDs was then selected to characterize representative motility behavior within the population without introducing visual or statistical bias from dense trajectory overlapping or overrepresented motion patterns.

Together, this approach allowed us to quantify heterogeneity in bacterial motility, identify dominant behavioral subtypes, and support subsequent investigations into how physical or chemical treatments alter single-cell motility phenotypes.

Text. S3 Fluorescence analysis

Di-8-ANEPPS is a voltage-sensitive, lipophilic dye whose fluorescence emission spectrum shifts in response to local membrane potential. Mechanistically, the dye integrates into the bacterial lipid bilayer, where its electronic state, and thus its emission profile, is influenced by the transmembrane electric field. Under hyperpolarized conditions (i.e., more negative membrane potential), the dye preferentially emits in the green region of the spectrum. As the membrane depolarizes (i.e., becomes less negative), the altered electric field promotes a shift in the dye's electronic configuration, resulting in increased orange

fluorescence. By exciting the dye at a fixed wavelength and capturing emissions in two spectral channels (green and orange), pixel-wise intensity ratios (orange/green) can be computed to quantify membrane potential. Since bacterial surface charge density modulates transmembrane potential, the orange/green fluorescence ratio provides a real-time, spatially resolved proxy for surface charge heterogeneity at the single-cell level.

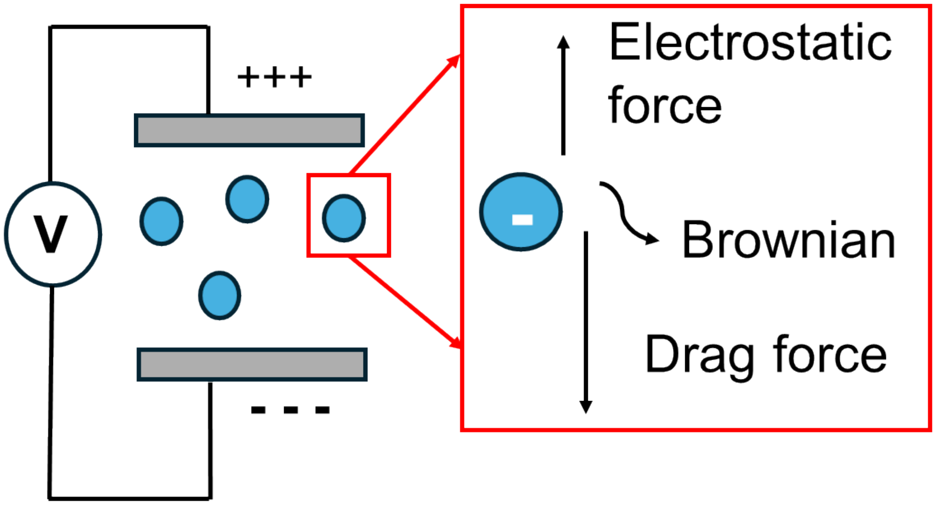

Figure. S1 An in-situ platform was developed by connecting a direct power source to a microchannel which contains bacteria. The zoomed view (right) illustrates the measurement principle: electrostatic force, drag force and Brownian motion.

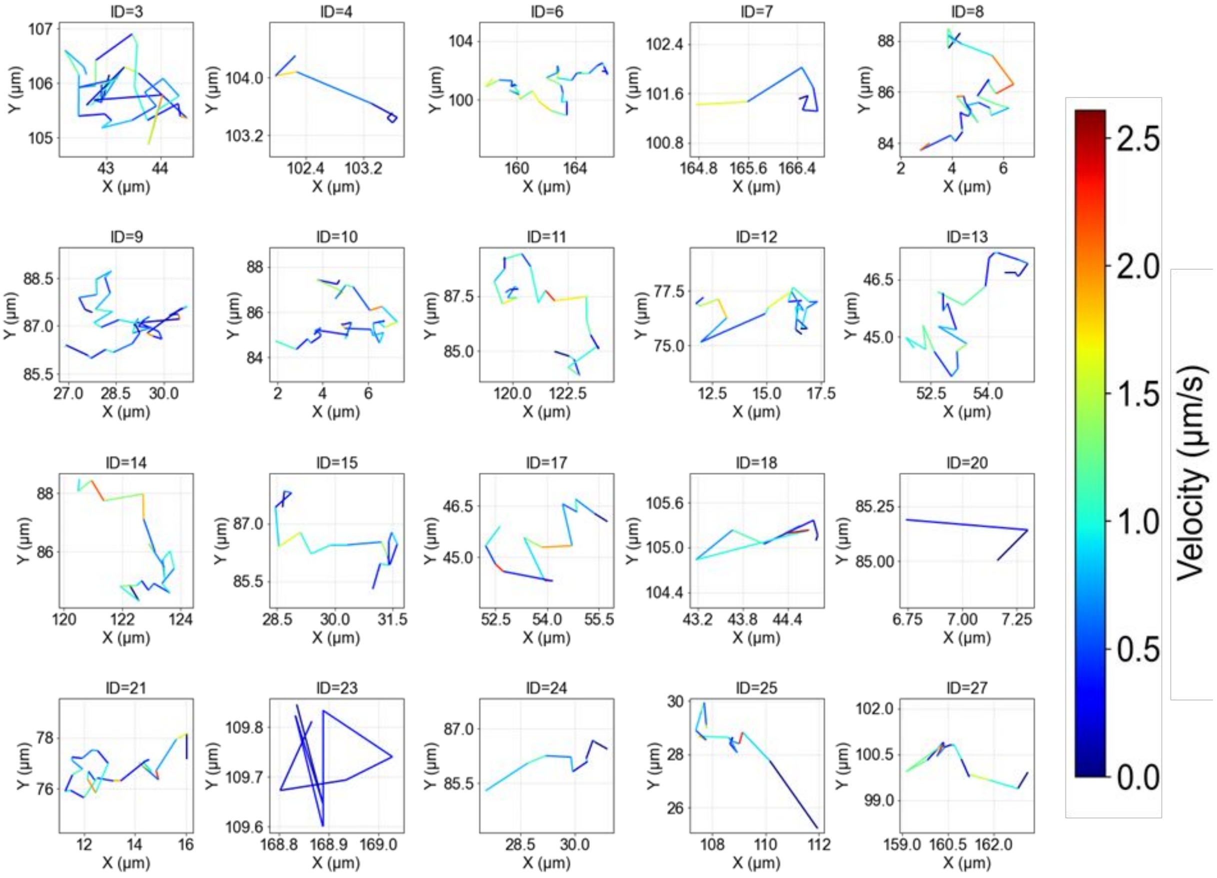

Figure. S2 Twenty representative single-cell tracks are shown as 2D paths (one bacterium per panel); line color encodes instantaneous velocity (color bar at right).

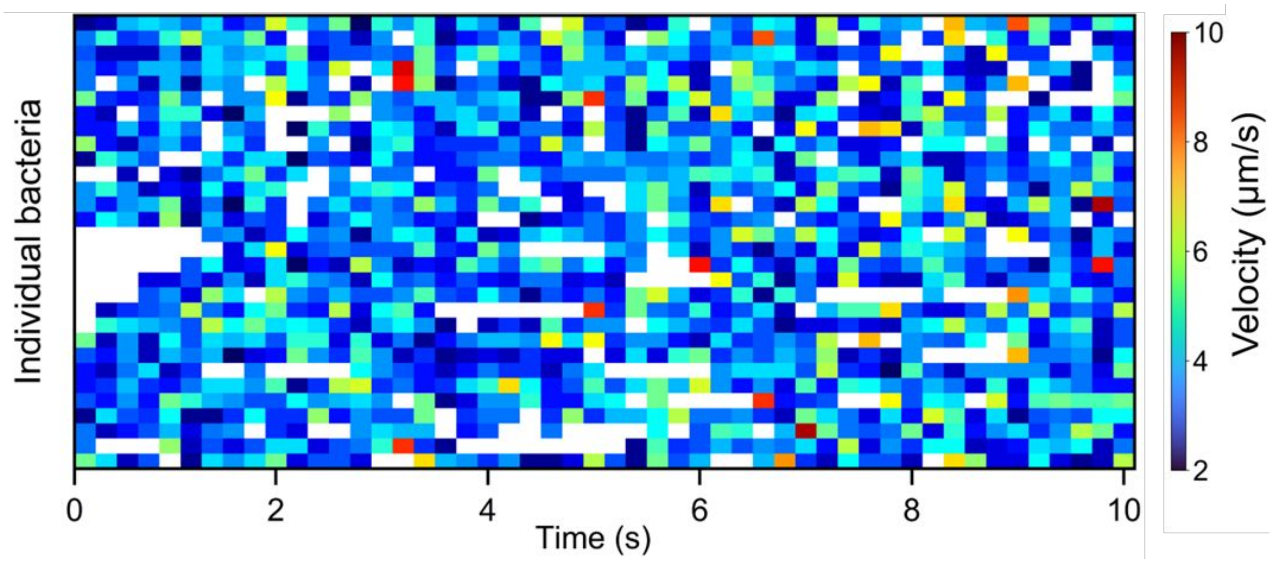

Figure. S3 Heat map of instantaneous velocities for individually tracked bacteria during a 10-s recording under a constant field. Each row is one bacterium (n=30); columns are time frames; color encodes velocity (scale at right). White pixels denote missing steps filtered out (collision or out-of-focus).

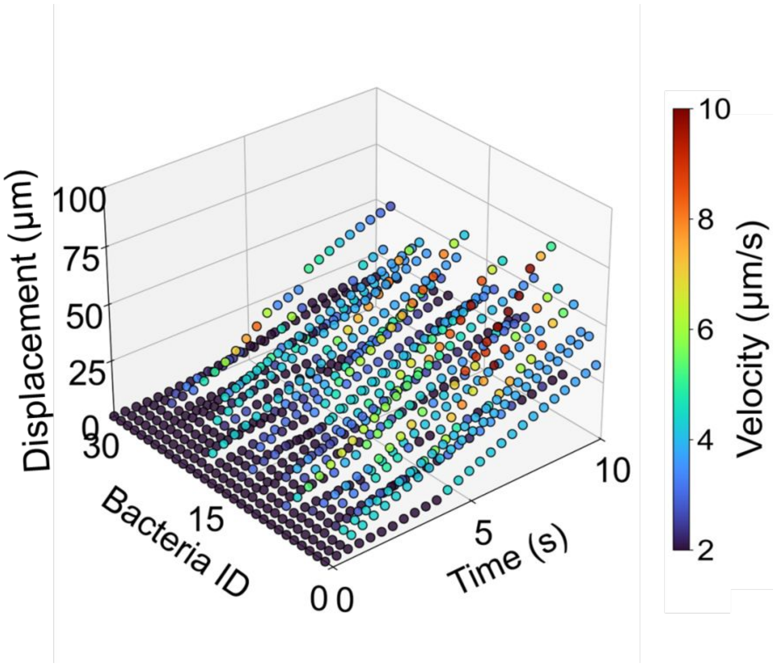

Figure. S4 Three-dimensional displacement where the x-axis represents the cell ID (30 samples selected as representative of all bacteria), the y-axis represents time, the z-axis reflects the displacement along the direction of the electric field, and the color bar represents the velocity in each frame.

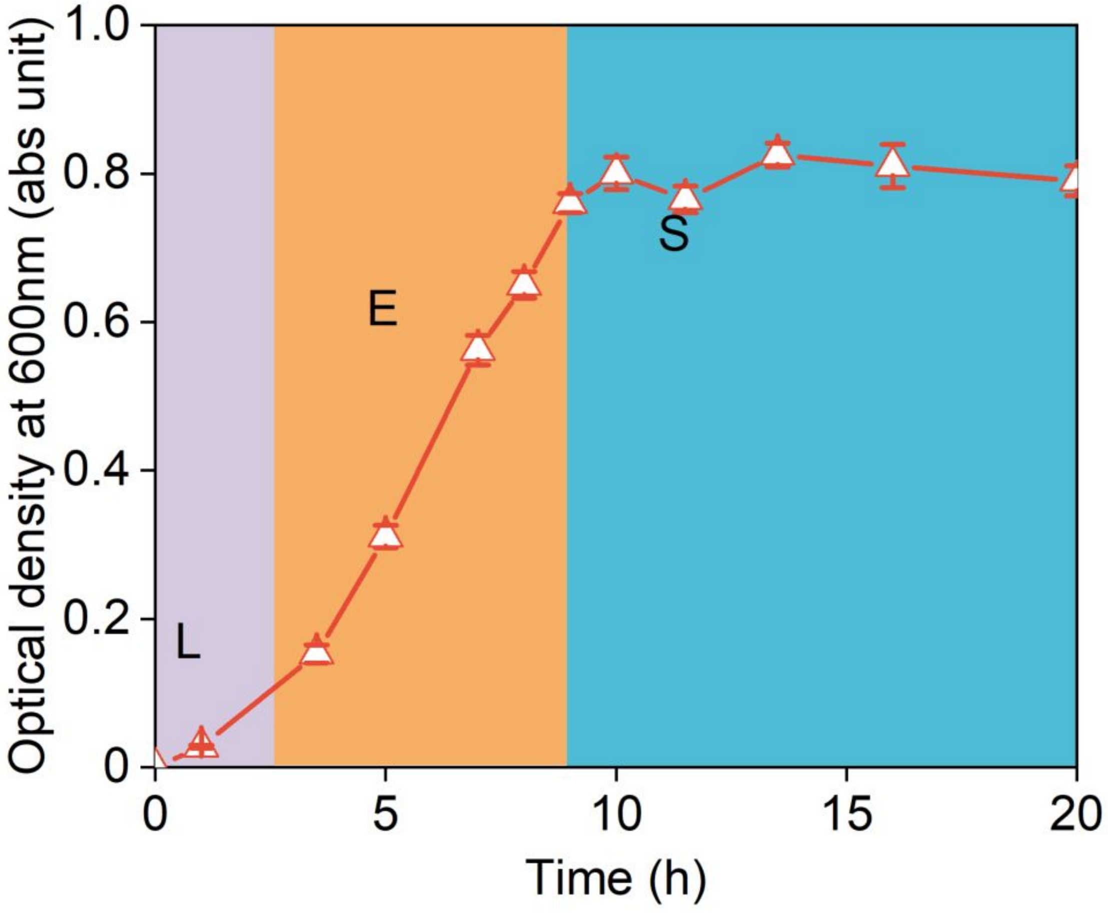

Figure. S5 Growth curve of the *Staphylococcus epidermidis* divided into three sections: L for lag phase, E for exponential phase, and S for stationary phase.

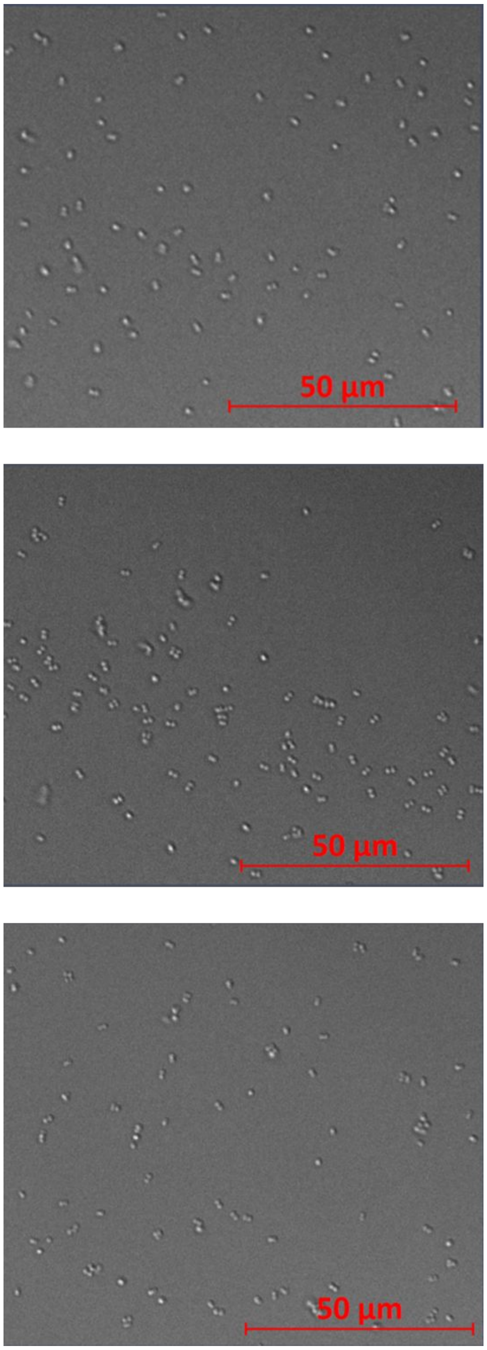

Figure. S6 Representative DIC image of bacteria in three growth phases. From top to bottom is lag, exponential, and stationary phase.

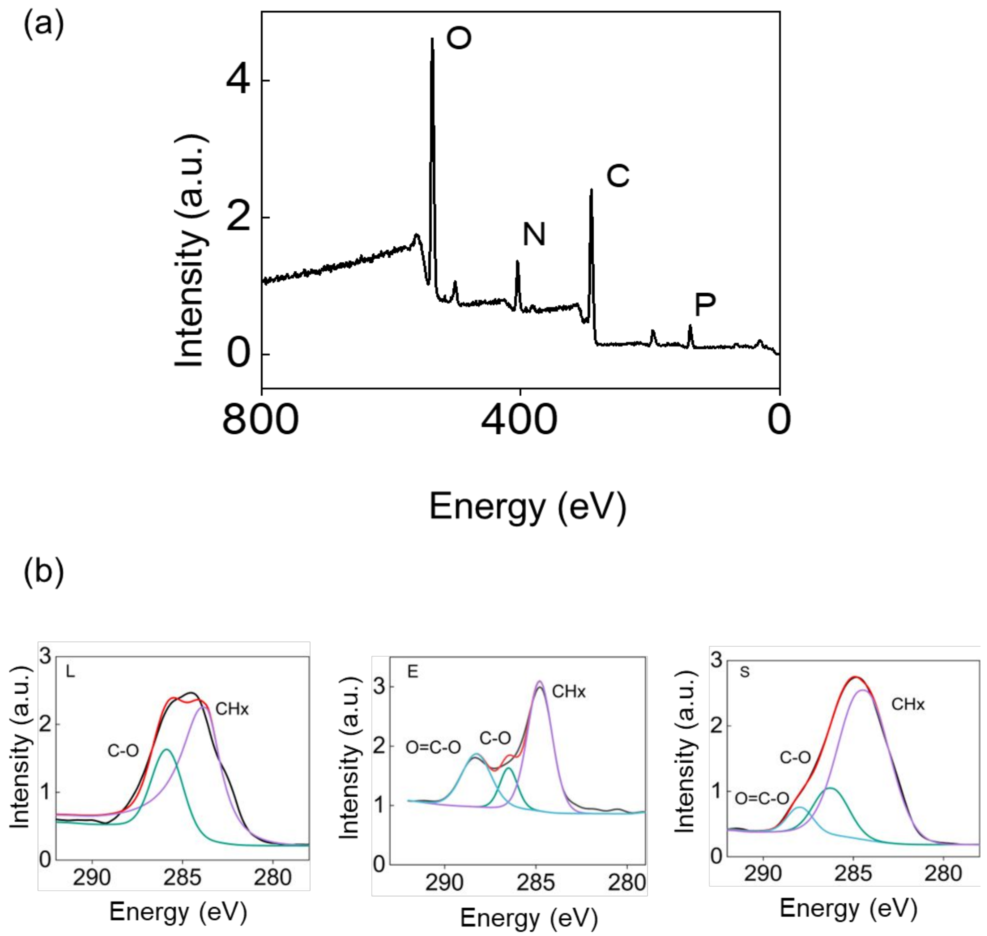

Figure. S7 X-ray photoelectron spectroscopy (XPS) analysis of different growth phases. (a) The survey map of XPS. (b) High-resolution C 1s XPS spectra for L, E, and S cells. Each spectrum is deconvoluted into three component peaks: oxidized carbon (O–C=O, 288.8 eV), C–O (286.5 eV), and hydrocarbons (C–H_x, 284.8 eV).

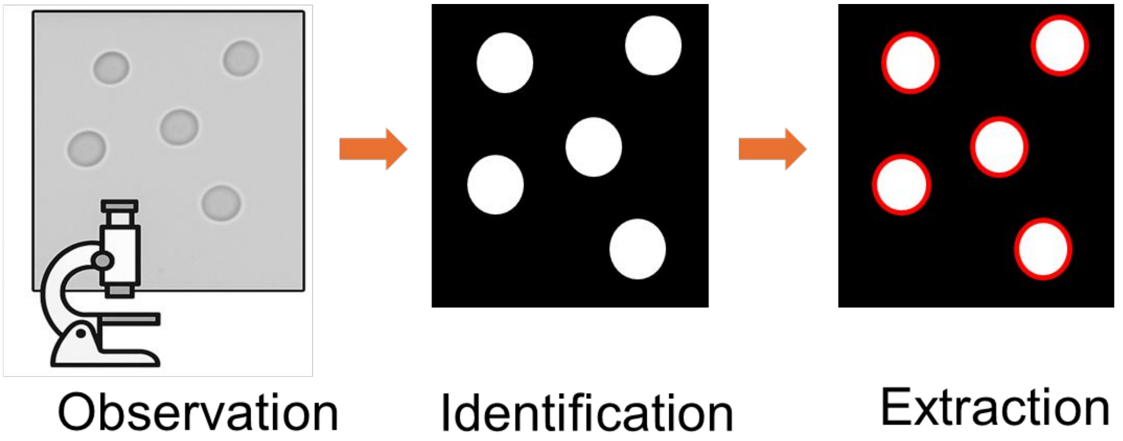

Figure. S8 Workflow for particle detection and tracking. Acquisition of microscopy frames (Observation), segmentation to identify cells (Identification), and ROI/centroid extraction (red) for quantitative tracking (Extraction).

(a)

(b)

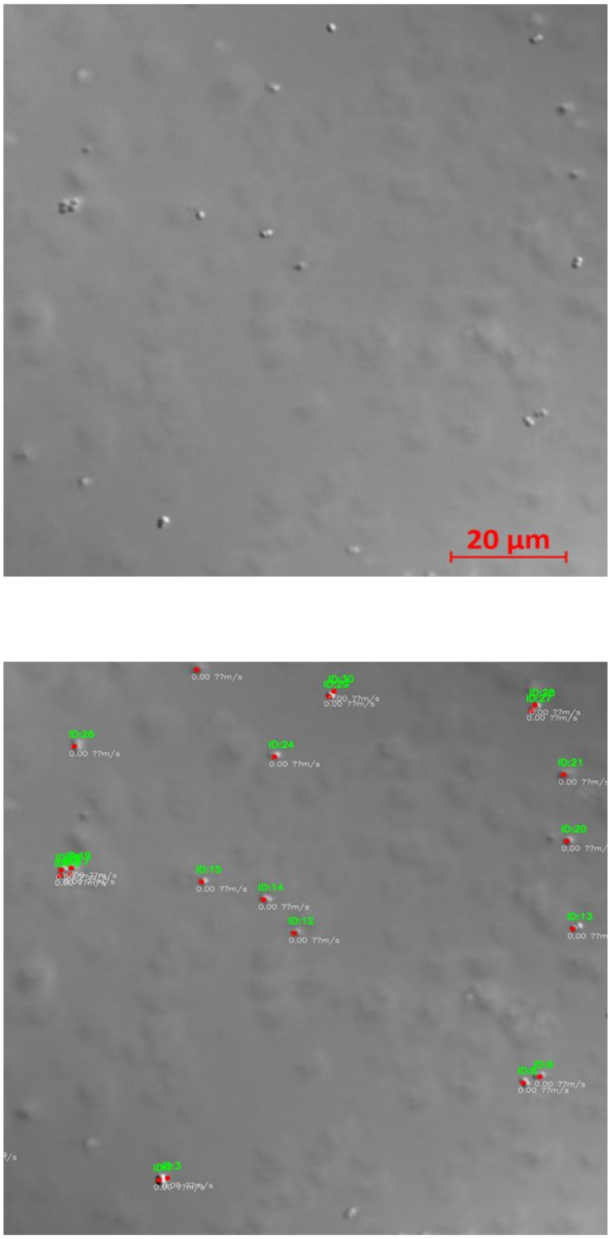

Figure. S9 Bacteria detection samples using Hungarian method. Bacteria were labelled with red dots and green IDs during the transport.

Supplementary code

Video track

```
import cv2

import numpy as np

import pandas as pd

import os

from scipy.spatial import distance

from scipy.optimize import linear_sum_assignment


PIXEL_TO_MICRON = 0.093

MAX_ASSOCIATION_DISTANCE = 150

MAX_LOST_FRAMES = 10

MIN_CONTOUR_AREA = 50

MIN_ENCLOSING_RADIUS = 2

directory_path = "./videos"


def detect_bacteria_positions(frame):

    gray = cv2.cvtColor(frame, cv2.COLOR_BGR2GRAY)

    blurred = cv2.GaussianBlur(gray, (9, 9), 0)

    thresh = cv2.adaptiveThreshold(blurred, 255, cv2.ADAPTIVE_THRESH_GAUSSIAN_C,
                                   cv2.THRESH_BINARY_INV, 11, 2)

    kernel = cv2.getStructuringElement(cv2.MORPH_ELLIPSE, (5,5))

    opened = cv2.morphologyEx(thresh, cv2.MORPH_OPEN, kernel, iterations=1)

    contours, _ = cv2.findContours(opened, cv2.RETR_EXTERNAL, cv2.CHAIN_APPROX_SIMPLE)

    points = []

    for cnt in contours:

        area = cv2.contourArea(cnt)
```

```
            if area > MIN_CONTOUR_AREA:
                (x, y), radius = cv2.minEnclosingCircle(cnt)
                if radius > MIN_ENCLOSING_RADIUS:
                    points.append((x, y))
    return points


class Track:
    def __init__(self, track_id, x, y, frame_idx):
        self.id = track_id
        self.x = x
        self.y = y
        self.distance = 0.0
        self.speed = 0.0
        self.last_frame = frame_idx
        self.lost_frames = 0

    def update(self, new_x, new_y, frame_idx, dt):
        dist_px = distance.euclidean((self.x, self.y), (new_x, new_y))
        dist_um = dist_px * PIXEL_TO_MICRON
        self.x = new_x
        self.y = new_y
        self.speed = dist_um / dt if dt > 0 else 0
        self.distance += dist_um
        self.last_frame = frame_idx
        self.lost_frames = 0


def associate_detections_to_tracks(tracks, detections):
```

```
    if not tracks or not detections:
        return [], list(range(len(tracks))), list(range(len(detections)))
    cost_matrix = np.zeros((len(tracks), len(detections)))
    for i, trk in enumerate(tracks):
        for j, (x, y) in enumerate(detections):
            cost_matrix[i,j] = distance.euclidean((trk.x, trk.y), (x, y))
    row_idx, col_idx = linear_sum_assignment(cost_matrix)
    matched = []
    unmatched_trk = list(range(len(tracks)))
    unmatched_det = list(range(len(detections)))
    for r, c in zip(row_idx, col_idx):
        if cost_matrix[r,c] <= MAX_ASSOCIATION_DISTANCE:
            matched.append((r, c))
            unmatched_trk.remove(r)
            unmatched_det.remove(c)
    return matched, unmatched_trk, unmatched_det


def main():
    video_files = [f for f in os.listdir(directory_path) if f.endswith('.avi')]
    for video_file in video_files:
        video_path = os.path.join(directory_path, video_file)
        cap = cv2.VideoCapture(video_path)
        if not cap.isOpened():
            print(f"Error opening video: {video_file}")
            continue
        fps = 5
        dt = 1 / fps
```

```
    frame_size = (
        int(cap.get(cv2.CAP_PROP_FRAME_WIDTH)),
        int(cap.get(cv2.CAP_PROP_FRAME_HEIGHT))
    )
    fourcc = cv2.VideoWriter_fourcc(*'XVID')
    output_path = os.path.join(
        directory_path,
        f"{os.path.splitext(video_file)[0]}_tracked.avi")
    out = cv2.VideoWriter(output_path, fourcc, fps, frame_size)
    tracks = []
    next_id = 1
    data_records = []
    frame_idx = 0
    while True:
        ret, frame = cap.read()
        if not ret:
            break
        detections = detect_bacteria_positions(frame)
        matched, unmatched_trk, unmatched_det = associate_detections_to_tracks(tracks,
detections)
        for trk_idx, det_idx in matched:
            x, y = detections[det_idx]
            tracks[trk_idx].update(x, y, frame_idx, dt)
        for trk_idx in unmatched_trk:
            tracks[trk_idx].lost_frames += 1
        for det_idx in unmatched_det:
            x, y = detections[det_idx]
            tracks.append(Track(next_id, x, y, frame_idx))
```

```
        next_id += 1
tracks = [trk for trk in tracks if trk.lost_frames <= MAX_LOST_FRAMES]
active_tracks = [trk for trk in tracks if trk.lost_frames == 0]
if active_tracks:
    speeds = [trk.speed for trk in active_tracks]
    max_idx = np.argmax(speeds)
    min_idx = np.argmin(speeds)
for i, trk in enumerate(tracks):
    if trk.lost_frames > 0:
        continue
    x_um = trk.x * PIXEL_TO_MICRON
    y_um = trk.y * PIXEL_TO_MICRON
    x, y = int(trk.x), int(trk.y)
    color = (0, 0, 255)
    label = f"ID:{trk.id}"
    if len(active_tracks) > 1:
        if i == max_idx and speeds[max_idx] > 0:
            color = (255, 0, 255)
            cv2.putText(frame, "FASTEST", (x+10, y-20),
                        cv2.FONT_HERSHEY_SIMPLEX, 0.6, color, 2)
        elif i == min_idx and speeds[min_idx] < speeds[max_idx]:
            color = (255, 255, 0)
            cv2.putText(frame, "SLOWEST", (x+10, y+45),
                        cv2.FONT_HERSHEY_SIMPLEX, 0.6, color, 2)
    cv2.circle(frame, (x, y), 5, color, -1)
    cv2.putText(frame, label, (x-10, y-20),
                cv2.FONT_HERSHEY_SIMPLEX, 0.6, (0,255,0), 2)
```

```
                cv2.putText(frame, f"{trk.speed:.2f} µm/s", (x-10, y+20),
                            cv2.FONT_HERSHEY_SIMPLEX, 0.5, (255,255,255), 1)
                data_records.append({
                    "Frame": frame_idx,
                    "ID": trk.id,
                    "X": x_um,
                    "Y": y_um,
                    "Distance": trk.distance,
                    "Speed": trk.speed
                })
            out.write(frame)
            frame_idx += 1
        cap.release()
        out.release()
        df = pd.DataFrame(data_records)
        excel_path = os.path.join(
            directory_path,
            f"{os.path.splitext(video_file)[0]}_tracked.xlsx")
        df.to_excel(excel_path, index=False)
        print(f"Processed {video_file}: {len(data_records)} records saved.")

if __name__ == "__main__":
    main()
```

Data analysis

```
import pandas as pd

import numpy as np

import matplotlib.pyplot as plt

from matplotlib.collections import LineCollection

from matplotlib.colors import Normalize

from matplotlib.ticker import MaxNLocator, ScalarFormatter

import matplotlib as mpl

import math


mpl.rcParams['font.family'] = 'Arial'


EXCEL_PATH = "./data/example_tracked.xlsx"


def plot_trajectories_with_speed(df, cmap='viridis', vmin=None, vmax=None):
    if vmin is None:
        vmin = df['Speed'].min()
    if vmax is None:
        vmax = df['Speed'].max()

    fig, ax = plt.subplots(figsize=(8, 6))
    lc_for_colorbar = None

    for track_id, group_data in df.groupby('ID'):
        if len(group_data) < 30:
            continue
```

```
        group_data = group_data.sort_values(by='Frame')
        xvals = group_data['X'].values
        yvals = group_data['Y'].values
        speeds = group_data['Speed'].values

        if len(xvals) < 2:
            continue

        segments = [((xvals[i], yvals[i]), (xvals[i+1], yvals[i+1])) for i in range(len(xvals) - 1)]
        color_values = speeds[:-1]

        lc = LineCollection(segments, array=np.array(color_values), cmap=cmap,
                            norm=Normalize(vmin=vmin, vmax=vmax), linewidth=2)
        ax.add_collection(lc)
        lc_for_colorbar = lc

    if lc_for_colorbar is not None:
        cbar = fig.colorbar(lc_for_colorbar, ax=ax)
        cbar.set_label("Speed (µm/s)", fontsize=16)
        cbar.set_ticks(np.linspace(vmin, vmax, 5))
        cbar.ax.tick_params(labelsize=14)

    ax.set_xlabel("X (µm)", fontsize=16)
    ax.set_ylabel("Y (µm)", fontsize=16)
    ax.tick_params(axis='both', labelsize=14)
    ax.set_title("All Trajectories Colored by Speed", fontsize=16)
    ax.grid(True, linestyle='--', alpha=0.5)
```

```
    ax.set_xlim(0, 180)
    ax.set_ylim(0, 120)
    ax.xaxis.set_major_locator(MaxNLocator(4))
    ax.yaxis.set_major_locator(MaxNLocator(4))
    ax.xaxis.set_major_formatter(ScalarFormatter(useOffset=False))
    ax.yaxis.set_major_formatter(ScalarFormatter(useOffset=False))

    plt.tight_layout()
    plt.show()

def plot_trajectories_per_id(df, cmap='jet', vmin=None, vmax=None,
                             colorbar_width=0.02, colorbar_pad=0.02):
    if vmin is None:
        vmin = df['Speed'].min()
    if vmax is None:
        vmax = df['Speed'].max()

    valid_ids = [track_id for track_id, group_data in df.groupby('ID') if len(group_data) >= 30]
    if not valid_ids:
        print("No valid ID with >=10 points, skip subplots.")
        return

    num_tracks = len(valid_ids)
    ncols = int(math.ceil(math.sqrt(num_tracks)))
    nrows = int(math.ceil(num_tracks / ncols))
    fig, axes = plt.subplots(nrows, ncols, figsize=(5 * ncols, 4 * nrows), sharex=False, sharey=False)
    fig.subplots_adjust(wspace=0.4, hspace=0.4)
```

```
axes = np.atleast_1d(axes).flatten()
last_lc = None

for i, track_id in enumerate(valid_ids):
    ax = axes[i]
    gdata = df[df['ID'] == track_id].sort_values(by='Frame')
    xvals = gdata['X'].values
    yvals = gdata['Y'].values
    speeds = gdata['Speed'].values

    ax.set_title(f"ID={track_id}, nPoints={len(gdata)}", fontsize=18)
    ax.set_xlabel("X (µm)", fontsize=18)
    ax.set_ylabel("Y (µm)", fontsize=18)
    ax.tick_params(axis='both', labelsize=16)
    ax.xaxis.set_major_locator(MaxNLocator(3))
    ax.yaxis.set_major_locator(MaxNLocator(3))
    ax.xaxis.set_major_formatter(ScalarFormatter(useOffset=False))
    ax.yaxis.set_major_formatter(ScalarFormatter(useOffset=False))

    if len(xvals) < 2:
        continue

    segments = [((xvals[k], yvals[k]), (xvals[k+1], yvals[k+1])) for k in range(len(xvals) - 1)]
    color_values = speeds[:-1]

    lc = LineCollection(segments, array=np.array(color_values), cmap=cmap,
```

```
                                        norm=Normalize(vmin=vmin, vmax=vmax), linewidth=2)
            ax.add_collection(lc)
            ax.autoscale()
            ax.grid(True, linestyle='--', alpha=0.5)
            last_lc = lc

        for j in range(num_tracks, len(axes)):
            axes[j].set_visible(False)

        fig.subplots_adjust(right=1.0 - colorbar_width - colorbar_pad)
        cbar_ax = fig.add_axes([1.0 - colorbar_width, 0.2, colorbar_width, 0.6])
        if last_lc is not None:
            cbar = plt.colorbar(last_lc, cax=cbar_ax)
            cbar.set_label("Speed (μm/s)", fontsize=22)
            cbar.set_ticks(np.linspace(vmin, vmax, 5))
            cbar.ax.tick_params(labelsize=22)

        plt.show()

def main():
    df = pd.read_excel(EXCEL_PATH)
    plot_trajectories_with_speed(df, cmap='turbo', vmin=0, vmax=20)
    plot_trajectories_per_id(df, cmap='jet', vmin=0, vmax=20)

if __name__ == "__main__":
    main()
```
